# Nematode-Infected Mice Acquire Resistance to Subsequent Infection With Unrelated Nematode by Inducing Highly Responsive Group 2 Innate Lymphoid Cells in the Lung

**DOI:** 10.3389/fimmu.2018.02132

**Published:** 2018-09-19

**Authors:** Koubun Yasuda, Takumi Adachi, Atsuhide Koida, Kenji Nakanishi

**Affiliations:** ^1^Department of Immunology, Hyogo College of Medicine, Nishinomiya, Hyogo, Japan; ^2^Department of Otolaryngology–Head and Neck Surgery, Kyoto Prefectural University of Medicine, Kyoto, Japan

**Keywords:** trained immunity, innate immune memory, intestinal nematode, eosinophils, IL-33

## Abstract

The immune responses against helminths have been investigated individually, and it is well-established that infected hosts develop an immunological memory to resist reinfection by the same pathogen. In contrast, it is poorly understood how the host immune system responds to subsequent infection by unrelated parasites after elimination of the first infection. We previously reported that infection of mice with *Strongyloides venezuelensis* induces the accumulation of group 2 innate lymphoid cells (ILC2s) in the lung. Here, we demonstrated that *S. venezuelensis*-experienced (Sv-exp) mice became significantly resistant against infection by *Nippostrongylus brasiliensis. N. brasiliensis* infection induced enhanced accumulation of ILC2s and eosinophils with increased expressions of mRNA for Th2 cytokines in the lungs of Sv-exp mice. The resistance was dependent on ILC2s, and eosinophils but not on CD4^+^ T cells. Furthermore, pulmonary ILC2s in Sv-exp mice acquired a highly responsive “trained” phenotype; in response to *N. brasiliensis* infection, they rapidly increased and produced IL-5 and IL-13, which in turn induced the early accumulation of eosinophils in the lungs. IL-33 was required for the accumulation of ILC2s and the resistance of mice against *N. brasiliensis* infection but insufficient for the induction of trained ILC2s. In conclusion, animals infected with one type of lung-migratory nematodes acquire a specific-antigen-independent resistance to another type of lung-migrating nematodes, providing animals with the capacity to protect against sequential infections with various lung-migratory nematodes.

## Introduction

Soil-transmitted intestinal parasites cause one of the most prevalent neglected tropical diseases (NTD) (1). Soil-transmitted helminths infect approximately 1.7 billion people in developing countries, creating both health- and economics-related problems (1–4). Particularly, in tropical and subtropical regions, such as sub-Saharan Africa, the Americas, China, and East Asia, the distribution of many infectious diseases caused by parasitic nematodes, such as *Ascaris lumbricoides*, hookworm *(Ancylostoma duodenale and Necator americanus), Trichuris trichiura*, and *Strongyloides stercoralis*, overlap one another (1, 2, 5). People in these areas can be infected by multiple parasites concurrently or sequentially.

These soil-transmitted helminths are gastrointestinal nematodes, and they are divided into two groups based on the infection routes. *A. lumbricoides* and *T. trichiura* parasitize directly to the intestinal tract by oral infection, hookworm and *S. stercoralis* transcutaneously invade and once go to the lungs and then migrate to the intestinal tract to mature. For this reason, the latter is known to cause eosinophilic inflammation in the lungs (Loeffler syndrome).

To resist such intestinal nematodes infection, hosts generally develop Th2 immune responses, which induce IgG1 and IgE production, intestinal mastocytosis, systemic eosinophilia, goblet cell hyperplasia, and intestinal smooth muscle contraction (6–9). Among these various reactions, the response required for appropriate protection depends on the type of infecting parasite. In an experimental animal model, the induction of goblet cells, epithelial cell turnover, and smooth muscle contraction, all of which are induced by the action of Th2 cell-derived cytokines (IL-4 and IL-13), are indispensable for the rapid expulsion of *N. brasiliensis*, a gut-dwelling rodent nematode (10–12).

*Strongyloides venezuelensis*, another rodent nematode, is a counterpart of the human pathogen *S. stercoralis* (13). In contrast to the required responses for *N. brasiliensis* expulsion, cytokine-induced mastocytosis and antibody (Ab)/FcRγ-dependent mucosal mast cell (MMC) activation are important for the rapid expulsion of *S. venezuelensis* from the intestine (14–18). Since the host memorizes this particular Th2 type immune response, when the same species of nematode invades again, a stronger immune response occurs to reject the infection (19, 20).

In addition to Th2 cells, group 2 innate lymphoid cells (ILC2s) also play a crucial role in host defense by secreting a large amount of type 2 cytokines in response to any epithelial barrier disruption by a migrating nematode (21, 22). These cells require the cytokine IL-7 along with a few transcription factors, such as retinoic acid receptor-related orphan receptor alpha (RORα) and GATA-binding protein 3 (GATA3), for their development (23–25). After stimulation with epithelial cell-derived cytokines [e.g., IL-25, IL-33, and thymic stromal lymphopoietin (TSLP)] or the neuropeptide neuromedin U, ILC2s start to produce the Th2 type cytokines IL-5, IL-13, and IL-9 (26–29). They also secrete amphiregulin, a member of the epidermal growth factor family, which stimulates tissue repair (30).

To date, the immune responses against helminths have been investigated individually, and it is well-established that helminth-infected hosts develop an immunological memory to resist a reinfection by the same pathogen. In contrast, it is poorly understood how the host immune system responds to subsequent infection by an unrelated intestinal nematode after elimination of the first infection. The aim of this study is to investigate the effect of primary infection on secondary infection with a different species of intestinal nematode and analyze *S. venezuelensis*-infected hosts acquisition of specific-antigen-independent resistance to infection by subsequent unrelated lung-migrating nematode by the induction of trained ILC2s.

## Materials and methods

### Ethics statement

This study was carried out in accordance with the recommendations of the Regulations for Animal Experimentation in Hyogo College of Medicine, Hyogo College of Medicine Animal Experiment Committee. The protocol was approved by the Hyogo College of Medicine Animal Experiment Committee (No. A11-076, 13-009, 16-006).

### Animals

C57BL/6 mice were purchased from Charles River Laboratories Japan. Wistar rats, and Sprague-Dawley (SD) rats were purchased from SLC Japan. *Il33*^−/−^ mice were produced and reported previously (31). RORα^sg/j^ mice were obtained from Jackson Laboratory. Δ*dblGATA* mice were purchased from Jackson Laboratory and backcrossed for 11 generations into the C57BL/6 background. All mice were used at 6–11 weeks of age except for the donor mice for bone marrow transplantation experiment. To prepare Rora^sg/sg^ bone marrow chimera mice, recipient 6-week-old C57BL/6 wild-type (WT) mice were lethally irradiated (5.5 Gy × 2, 4 h interval). Donor bone marrow cells (3–10 × 10^6^ cells per body) prepared from 3- to 4-week-old RORα^sg/+^ or RORα^sg/sg^ mice were transferred intravenously into recipients. Chimera mice were used for experiments from 12 weeks after transplantation. The numbers of mice used in each experiment were indicated in each figure legend.

### Reagents

Fluorescent-labeled antibodies for CCR3 (FAB729F, FITC, #83101) was purchased from R&D Systems, Siglec F (552126, PE, E50-2440) was purchased from BD Biosciences, for Gr-1 (108408, PE, RB6-8C5), CD45 (103134, brilliant violet 421, 30F11), Thy1.2 (140306, pacific blue, 53-2.1), Sca-1 (108142, AlexaFluor700 or 108112, APC, D7), CD3 (100308, PE or 100306, FITC, 145-2C11), CD4 (100512, PE or 100540, PerCP-Cy5.5, RM4-5), CD8 (100708, PE, 53-6.7), CD11b (101208, PE or 101206, FITC, M1/70), CD19 (115508, PE, 6D5), NK1.1 (108708, PE or 108706, FITC, PK136), B220 (103206, FITC, RA3-6B2), and IL-5 (504306, APC, TRFK5) were purchased from BioLegend, for IL-13 (12-7133-41, PE, eBio13A), GATA3 (12-9966-42, PE, TWAJ), and IL-25R (12-7361-82, PE, MUNC33) were purchased from eBioscience, for T1/ST2 (101001F, FITC or 101001B, biotin, DJ8) was purchased from MD Biosciences, and for IgE (1130-09L, PE, 23G3) was purchased from Southern Biotechnology Associates Inc. PerCP-Cy5.5-streptavidin was purchased from BioLegend (405214). Recombinant mouse IL-7 (407-ML) and IL-25 (1399-IL) were purchased from R&D Systems. Ionomycin and phorbol 12-myristate 13-acetate (PMA) were purchased from Sigma-Aldrich Japan. Recombinant human IL-33 (rhIL-33) was made by Hokudo Co., Ltd. as previously described (32).

### IL-33 administration

Mice were intranasally administered with 30–50 μl containing 100 ng of rhIL-33 or PBS under anesthesia, and right lung cells (prepared as described below), BALF cells from the left lung, and peripheral blood leukocytes (PBLs) were collected at 0, 7, 14, 21, and 28 days post-IL-33 administration.

### Helminth infections

*S. venezuelensis* was provided by Dr. H. Maruyama (University of Miyazaki) and maintained in male Wistar rats in our laboratory (15). Mice were subcutaneously inoculated in the back with 4,000–5,000 *S. venezuelensis* L3. *N. brasiliensis* was provided by Dr. M. Yamada (Kyoto Prefectural University of Medicine) and maintained in male SD rats (33). Mice were subcutaneously inoculated in the inguinal region with 500 *N. brasiliensis* L3. The degrees of infection of individual mice were monitored by counting the number of eggs excreted in feces daily (eggs/g of feces). For *S. venezuelensis* eggs, we counted eggs by direct smear method. In brief, feces fleshly passed from each mouse were weighed and suspended in a known volume of water in centrifuge tubes. The eggs were counted using a microscope (13). For *N. brasiliensis* eggs, we counted eggs after concentration with formalin-ether sedimentation method with minor modification (34). In brief, feces were weighed and dissolved in water, filtered through one layer of gauze, and centrifuged at 700 g for 3 min, the sediment was suspended with 10% formalin, after 20 min, ether was added, and shaken for 30 s, and the tube was again centrifuged and the pellet was recovered. Lung cells (prepared as described below), BALF cells, and PBLs were collected at 0, 7, 14, 21, and 28 days after *S. venezuelensis* infection.

To quantify lung stage larvae, mice were euthanized by cervical dislocation 2 days after infection, the whole lung from each mouse was harvested, minced, and incubated on a steel mesh put on a beaker containing 100 ml of PBS for 2 h at 37°C. Worms that emerged from the lung tissue were counted using a microscope (40x). To assess intestinal worm burdens, the whole small intestine from each infected mouse at the indicated day was removed and incubated on a steel mesh as above. The number of worms that emerged from the intestinal tissue was counted.

For IL-5 neutralization, mice received an intraperitoneal administration of 100 μg of anti-IL-5 monoclonal antibody (TRFK5) (35) at day 0 and day 2 post-infection with 500 *N. brasiliensis* L3. For CD4 depletion experiments, mice received an intraperitoneal administration of 500 μg of anti-CD4 monoclonal antibody (GK1.5) 1, 5, and 9 days before *N. brasiliensis* infection.

### Intraduodenal implantation of adult worms

Adult worms were prepared from the intestines of SD rats inoculated with 4,000 infective larvae 7 days previously. The adult worms were suspended in 200 μl of PBS and injected into the duodenum of recipient mice (175 worms/mouse) under anesthesia using a 1-ml syringe with an 18-gauge angiocath (BD Biosciences). Sixteen hours later, the mouse intestines were removed, cut open longitudinally, and incubated at 37°C in PBS for 2 h. The number of worms that emerged from the intestines was counted. For histological analysis, intestine specimens were sampled at about 6–12 cm distal from pylorus, fixed with 4% (wt/vol) paraformaldehyde (PFA) and embedded in paraffin. Those tissues were sectioned at 8-μm thickness and deparaffinized sections were stained with hematoxylin and eosin.

### Preparation of lung cells

At the indicated time points after infection, the whole or right lungs were prepared for cell analysis after perfusing the mice via the right ventricle with PBS under anesthesia. Whole or right lungs were infused with complete medium [RPMI 1640 supplemented with 10% fetal bovine serum, 2-ME (50 μM), L-glutamine (2 mM), penicillin (100 U/ml), and streptomycin (100 μg/ml)] containing collagenase (400 U/ml) and DNase I (10 μg/ml). The samples were then minced and digested for 60 min at 37°C. Cell suspensions were filtered using a cell strainer, and red blood cells were lysed. For sorting ILC2s, lung cells were stained with CD45, Thy1.2, ST2, and Sca-1 in addition to lineage (Lin) markers (CD3, CD19, CD11b, NK1.1). CD45^+^Lin^−^Thy1.2^+^Sca-1^+^ST2^+^ cells were sorted as ILC2s using a FACS Aria III (BD Biosciences).

### Bronchoalveolar lavage (BAL)

BAL was performed on the left lung with three aliquots of 0.5 ml of PBS per mouse, with a ligature of the right main bronchus by forceps, after perfusing the mouse via the right ventricle with PBS under anesthesia. The resulting BALF was examined for cytokine concentrations by ELISA using an ELISA Duo set for mouse IL-5 and IL-13 (R&D systems), and BALF cells were analyzed using flow cytometry.

### Flow cytometry

BALF cells, lung cells, and PBLs were stained with antibodies for surface antigens and analyzed using a FACSCalibur, LSRFortessa X-20 (BD Biosciences), or SP6800 (SONY) flow cytometer. Cells were classified as indicated in each figure legend. For intracellular cytokine staining, lung cells were stimulated with 50 nM PMA and 1 μM ionomycin for 4 h in the presence of brefeldin A or with 10 ng/ml IL-7, 100 ng/ml IL-25, and 100 ng/ml IL-33 for 24 h (brefeldin A was added for the last 6 h). Cells were stained with eBioscience Fixable viability dye eFlour506 (Thermo Fisher) and with antibodies against surface antigens. Fixation, permeabilization, and intracellular cytokine staining were performed using an eBioscience Foxp3/Transcription factor staining buffer kit and antibodies for IL-5 and IL-13 and were analyzed using a SP6800 flow cytometer.

### Quantitative RT-PCR (Q-PCR)

Total RNA was extracted with a RNeasy Mini Kit (Qiagen), and cDNA was synthesized using RevertraAce (TOYOBO). The expression levels of genes were quantified with TaqMan Gene Expression Assays (Applied Biosystems). The results are shown as the relative expression standardized against the expression of the gene encoding β-actin. Specific primers and probes used for quantitative RT-PCR were *Il33* (Mm00505403_m1), *Il5* (Mm00439646_m1), *Il13* (Mm00434204_m1), *Epx* (Mm00514768_m1), and *actin-b* (4352933E) (Applied Biosystems).

### Statistics

All data are shown as the mean ± S.D. The numerical data were analyzed using either a Student's t-test, one-way ANOVA with Bonferroni's *post-hoc* test, or two-way ANOVA with Bonferroni's *post-hoc* test using Prism (GraphPad Software), and *p*-values of less than 0.05 were considered significant. In all figures, ^*^p < 0.05, ^**^p < 0.01, ^***^p < 0.001, and ^****^p < 0.0001.

## Results

### *Strongyloides venezuelensis* infection-experienced mice develop an enhanced lung inflammation and show a substantial resistance to *nippostrongylus brasiliensis* infection

In the life cycles of *S. venezuelensis* and *N. brasiliensis*, the third stage larvae (L3) infect the skin and migrate to the lung before they mature in the intestine (36, 37). Within the lung, they induce severe inflammatory changes (i.e., mouse Loeffler syndrome), characterized by severe eosinophilic infiltration and goblet cell hyperplasia via the action of Th2 cytokines mainly from IL-33-activated ILC2s (31). However, mice that have been previously infected with these nematodes inhibit re-infection with the same pathogen by preventing larval migration to the lung via acquired immunity (38, 39).

**Figure 1:**
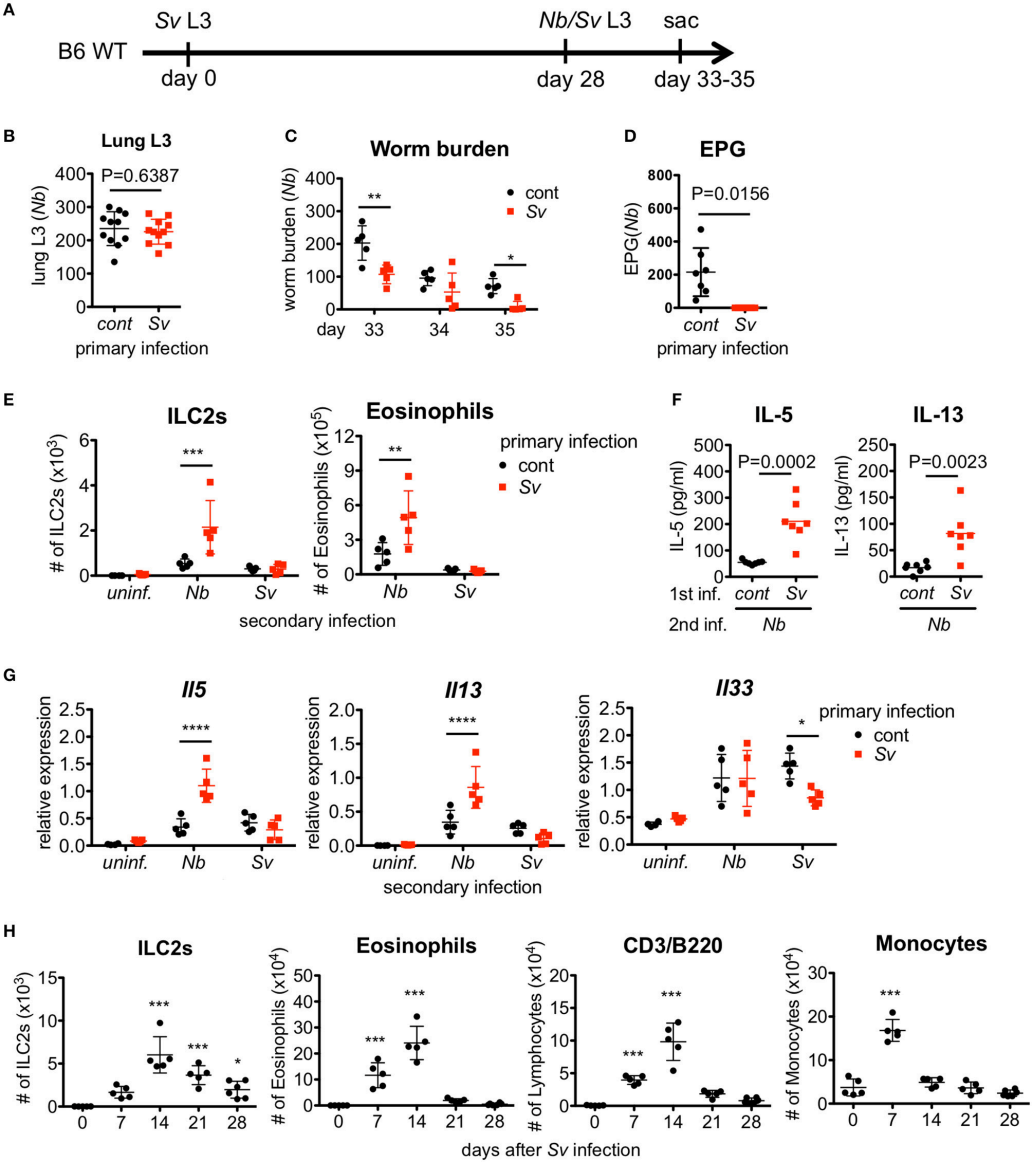
*Strongyloides venezuelensis*-experienced mice develop an increased pulmonary inflammation and a resistance against *Nippostrongylus brasiliensis* infection. **(A)** Experimental workflow for sequential nematode infection. *S. venezuelensis* (Sv)-infected or uninfected mice were inoculated with 500 *N. brasiliensis* (Nb) L3 at day 28. B6; C57BL/6, WT; wild type, sac; sacrificed. **(B)** The number of lung stage *N. brasiliensis* larva. Two days after *N. brasiliensis* infection, migrating larva were isolated from the lungs and counted (*n* = 11). cont, control (uninfected at day 0). Pooled data from two independent experiments are shown (Mean ± SD). **(C)** The numbers of worms in the intestine were counted at indicated days (*n* = 5). **(D)** The numbers of eggs per gram feces (EPG) from each group at day 7 post-*N. brasiliensis* infection (*n* = 7). **(E–G)** The numbers of eosinophils and ILC2s **(E)**, the amounts of IL-5 and IL-13 in the BALF **(F)**, and the *Il5, Il13*, and *Il33* mRNA expression levels in the lungs as analyzed by Q-PCR **(G)** from day 35 are shown (*n* = 4–5). uninf.; uninfected at day 28. Statistical analyses were conducted using two-way ANOVAs with Bonferroni *post-hoc* tests **(B,E,G)**, Wilcoxon matched-pairs signed rank tests, and two-tailed **(C)** or unpaired t-tests **(F)**. Data are representative of two independent experiments. **(H)** C57BL/6 WT mice (*n* = 5–6) were infected with 5000 L3 *S. venezuelensis* at day 0. The numbers of ILC2s, eosinophils, CD3^+^/B220^+^ cells, and monocytes among the BALF cells at the indicated days were analyzed by flow cytometry (FACScalibur). Cell populations were defined as follows: ILC2s, FSC^lo^SSC^lo^Lin(CD3, CD4, CD8, CD19, NK1.1, IgE, Gr-1, siglecF)^−^Sca-1^+^ST2^+^; Eosinophils, CD45^+^CD3^−^B220^−^CCR3^+^, CD3/B220: CD45^+^CCR3^−^CD3^+^/B220^+^; and Monocytes, CD45^+^Autofluorescence^high^. Data were compared with day 0 data by one-way ANOVA. Data are representative of two independent experiments.

To examine the effect of prior nematode infection on subsequent infection with a different nematode, we subcutaneously infected 6-week-old naive WT C57BL/6 mice with L3 of *S. venezuelensis*. The infected WT mice normally expel this parasite after 2 weeks due to the action of mast cells (16, 17). At 4 weeks after the primary infection, *S. venezuelensis*-experienced (Sv-exp) or age-matched uninfected control mice were infected with L3 of *N. brasiliensis*, which is another species of lung migrating intestinal nematode, or were re-infected with L3 of *S. venezuelensis* (Figure 1A). First, we confirmed that comparable numbers of *N. brasiliensis* larvae migrated to the lungs in control and Sv-exp mice (Figure 1B), indicating the occurrence of normal larval migration from the skin to the lungs in Sv-exp mice. We found that, at days 5 and 7 after *N. brasiliensis* infection (day 33 and day 35 after *S. venezuelensis* infection), Sv-exp mice had significantly lower worm burdens compared with *S. venezuelensis*-uninfected control mice (Figure 1C). Further, we were unable to detect eggs in the feces from Sv-exp mice (Figure 1D), indicating that Sv-exp mice acquired resistance to infection by *N. brasiliensis*.

Because host animals expel nematodes via the effects of Th2 cytokines, we analyzed the cytokine expression levels in the intestine. Sv-exp mice did not show an enhancement in cytokine expression compared with control mice (Figure S1A), indicating that the resistance to infection by *N. brasiliensis* is not caused by a severe inflammatory response in the intestine. Furthermore, we detected comparable worm burdens in both groups when we inoculated *N. brasiliensis* adult worms directly into the intestine (Figure S1B). They migrated into the intervillous space of jejunum (Figure S1C), suggesting that the intestines in Sv-exp animals were conducive to parasitic infection.

To investigate the immune responses in the lung, we analyzed the accumulation of inflammatory cells in the bronchoalveolar lavage fluid (BALF). Following *N. brasiliensis* infection, Sv-exp mice showed larger accumulations of ILC2s and eosinophils in the lung alveolus compared with control (no primary infection) mice (Figure 1E and Figure S2). As ILC2s are known to produce large amounts of the Th2 cytokines IL-5 and IL-13 (40, 41), it is not surprising that we detected higher levels of IL-5 and IL-13 in the BALF from *N. brasiliensis*-infected Sv-exp mice compared with control mice (Figure 1F). Accordingly, pre-infection with *S. venezuelensis* also enhanced the expression of mRNA for IL-5 and IL-13 in the lungs (Figure 1G). In contrast, reinfection with *S. venezuelensis* did not induce such hyper-responses (Figures 1E,G), probably due to the prevention of larval migration to the lung via acquired immunity provoked by the primary infection (39).

Since *S. venezuelensis* infection induces an activation of ILC2s that is IL-33-dependent (31), we analyzed the level of *Il33* mRNA expression in Sv-exp and *S. venezuelensis* infection-naive mice. The experience of *S. venezuelensis* infection did not influence the expression of *Il33* mRNA (Figure 1G), either before or after *N. brasiliensis* infection, indicating that the increase in the number of ILC2s was not caused via an enhancement of IL-33 production.

To determine if these results are specific responses to *S. venezuelensis* infection, we conducted the reverse experiment, beginning with *N. brasiliensis* as the primary infection. Notably, the experience of *N. brasiliensis* infection similarly reduced the number of eggs per gram of feces in *S. venezuelensis*-infected mice and increased the number of eosinophils in the BALF (Figure S3).

We were able to detect a protective effect of prior *S. venezuelensis* infection even 3 months later (Figures S4A,B). The numbers of ILC2s and eosinophils tended to be enhanced by *N. brasiliensis* infection 3 months after *S. venezuelensis* infection, though these differences failed to reach statistical significance (Figure S4C). Overall, the protective effect induced by experiencing an *S. venezuelensis* infection lasts at least 3 months after the first infection.

The above data suggest that the lung is the important site for protection against a subsequent infection by other pulmonary migratory nematode. To investigate the changes in the lungs after *S. venezuelensis* infection, we analyzed cells in the BALF at different time points after *S. venezuelensis* infection. In agreement with our previous report, *S. venezuelensis* infection resulted in a rapid increase in the number of inflammatory cells in the BALF (Figure 1H) (31). After the initial expansion, the numbers of ILC2s, eosinophils, and lymphocytes all peaked at day 14. Around day 12, adult worms were expelled from the intestine in WT mice (16, 17). Eosinophil and macrophage populations declined to their basal levels at day 21 and day 14, respectively, indicating the resolution of inflammation. In contrast, many ILC2s persisted in the BALF, even at day 28. These data suggest that the ILC2s remaining in the lungs after infection contribute to protection against a subsequent infection by other pulmonary migratory nematode.

### ILC2s contribute to the resistance against *nippostrongylus brasiliensis* infection in sv-exp mice

**Figure 2:**
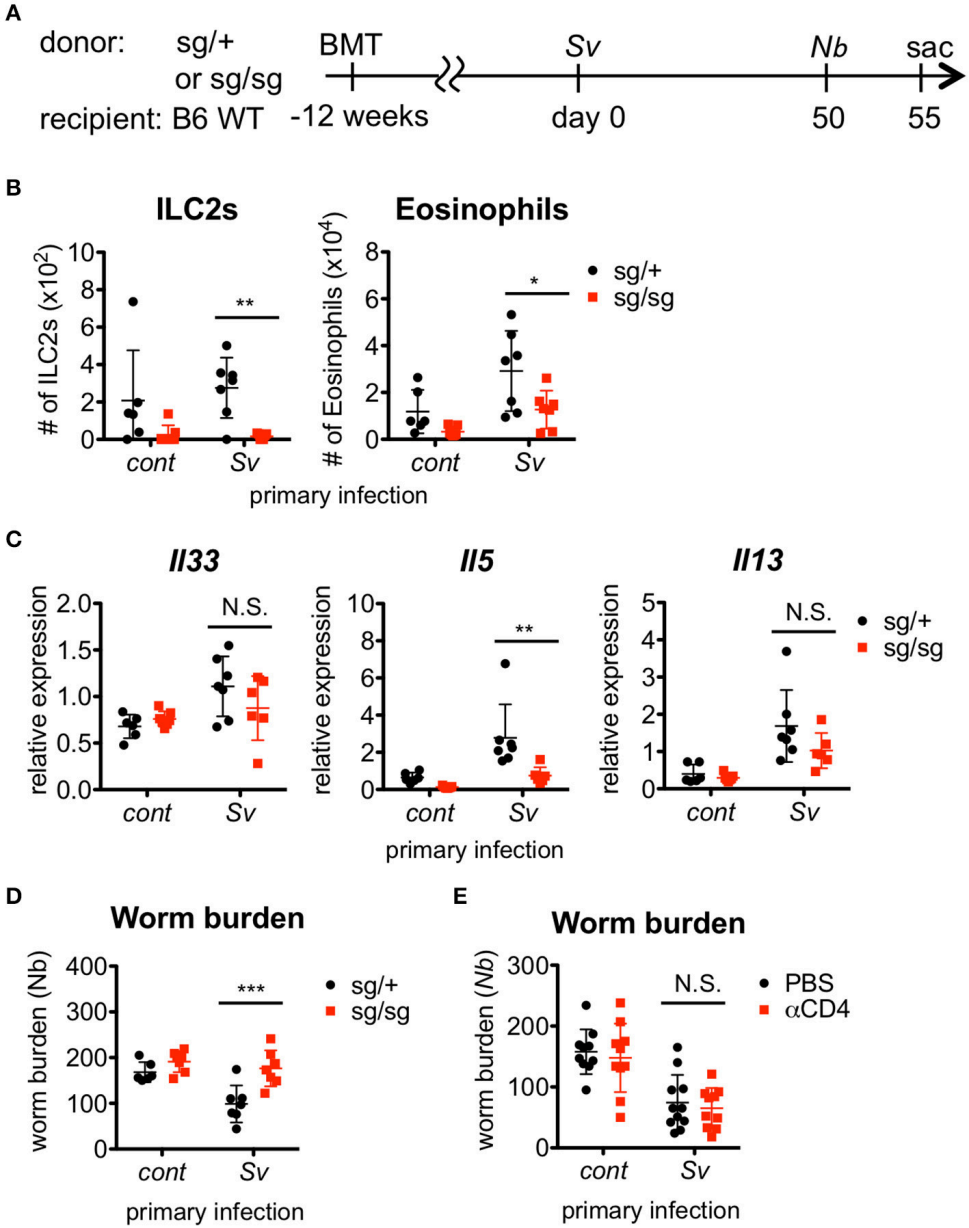
ILC2s are critical for the enhanced inflammation and the protection against *Nippostrongylus brasiliensis* infection. **(A)** Experimental workflow for sequential nematode infection in bone marrow chimera mice. BMT; bone marrow transplantation, sac; sacrificed. *S. venezuelensis*-infected (Sv) or uninfected (cont) mice (*Rora*^sg/+^, *n* = 6–7; *Rora*^sg/sg^, *n* = 7) were infected with *N. brasiliensis* (Nb). Five days after *N. brasiliensis* infection, **(B)** ILC2s and eosinophils among the BALF cells were analyzed by flow cytometry (LSRFortessa). Cell populations were defined as follows: ILC2s, FSC^lo^SSC^lo^CD45^+^CD4^−^Lin(CD3, CD8, CD19, NK1.1, IgE, siglec F, Gr-1)^−^Sca-1^+^ST2^+^ and Eosinophils, CD45^+^CD11c^lo^CD3^−^B220^−^CCR3^+^. **(C)** *Il33, Il5*, and *Il13* mRNA expression levels in the lungs were examined. **(D)** The numbers of worms in the intestine were counted at day 5 post-*N. brasiliensis* infection. Data are representative of two independent experiments. **(E)** *S. venezuelensis*-infected or uninfected WT mice were infected with *N. brasiliensis* after anti-CD4 Ab treatment (αCD4) as in Figure S5. The numbers of worms in the intestine were counted at day 5 after *N. brasiliensis* infection (*n* = 10–11). Pooled data from two independent experiments are shown (Mean ± SD).

Since Sv-exp mice were found to have an increased number of ILC2s in the lungs, we next examined whether ILC2s are required for the hyper-responses in the lung and the lower *N. brasiliensis* worm burden that occur in these mice. To investigate the role of ILC2s, we made ILC2-deficient *Rora*^sg/sg^ bone marrow chimera mice and infected them with nematodes (Figure 2A). First, we confirmed the absence of ILC2s in *Rora*^sg/sg^ chimera mice (Figure 2B). Notably, the eosinophil accumulation was reduced in ILC2-deficient mice compared with ILC2-sufficient *Rora*^sg/+^ control chimera mice (Figure 2B). The expression levels of Th2 cytokines, which are major products of ILC2s, were also decreased in the lungs of *Rora*^sg/sg^ chimera mice (Figure 2C), though low levels of cytokine expression remained. Probably other Th2 cytokine-producing cells, such as T cells, mast cells and basophils, cause the remaining cytokine expressions. Furthermore, *S. venezuelensis*-infection-induced resistance against *N. brasiliensis* was impaired in ILC2-deficient mice (Figure 2D), suggesting that the protective phenotype of Sv-exp mice requires RORα-dependent innate lymphoid cells.

In addition to ILC2s, *S. venezuelensis* infection induces strong Th2 differentiation of CD4^+^ helper T cells, as a general result of nematode infection (7, 42, 43). To examine the contribution of Th2 cells to the enhanced eosinophil accumulation and protection against subsequent *N. brasiliensis* infection, we depleted CD4^+^ T cells by treating Sv-exp mice with an anti-CD4 Ab before *N. brasiliensis* infection (Figure S5A). The resistance against *N. brasiliensis* infection and the alveolar accumulation of ILC2s and eosinophils in Sv-exp mice were not affected by the absence of CD4^+^ T cells (Figure 2E and Figure S5B), indicating that Th2 cells are dispensable for these responses. These data demonstrate that Sv-exp mice acquired resistance to *N. brasiliensis* via ILC2-dependent innate immune responses.

### Eosinophils are essential for the protection of sv-exp mice from *nippostrongylus brasiliensis* infection

**Figure 3:**
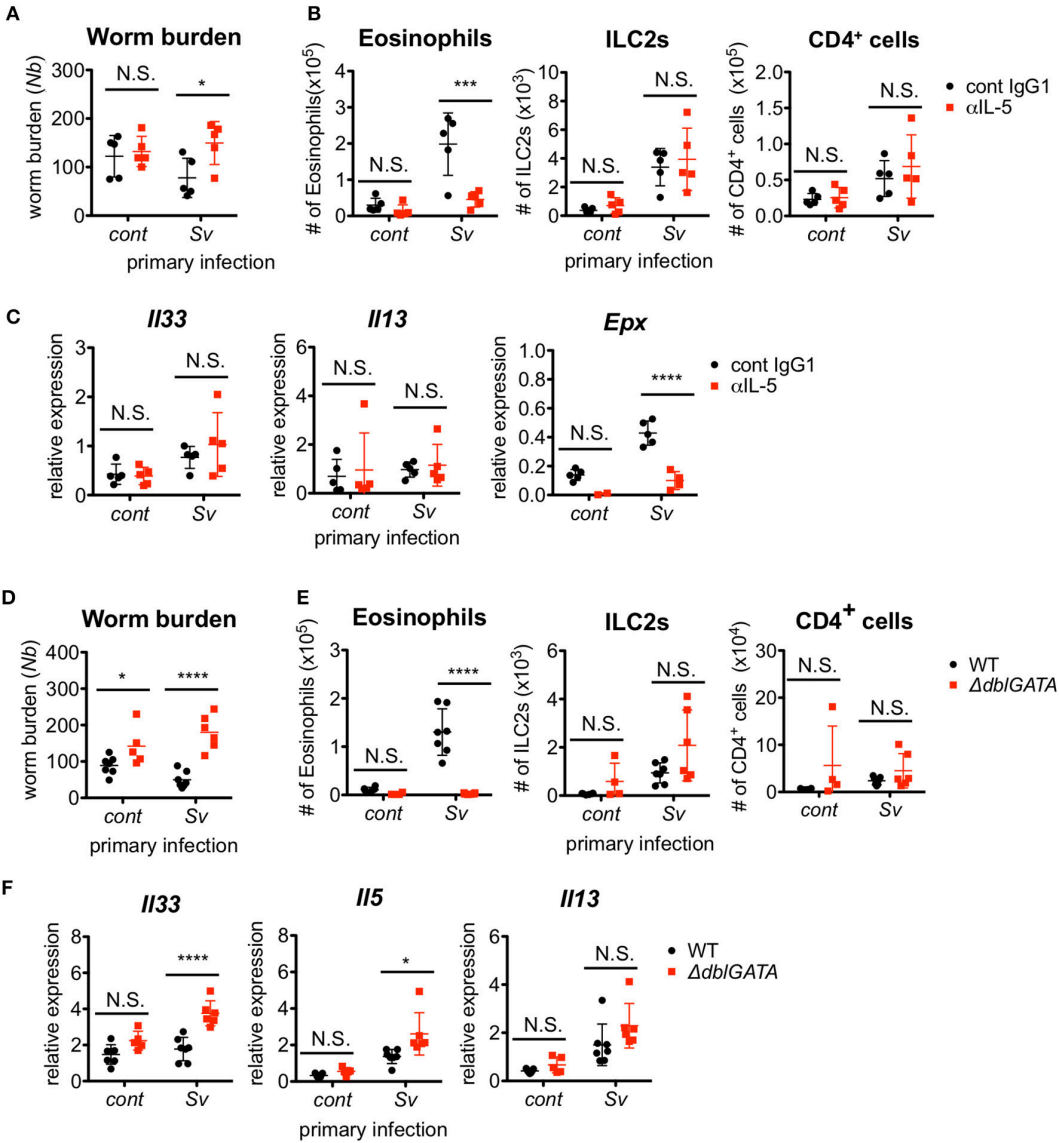
Eosinophils are essential for resistance against *Nippostrongylus brasiliensis*. **(A–C)** *S. venezuelensis*-infected (Sv) or uninfected (cont) mice (*n* = 5) were treated with anti-IL-5 Ab (TRFK5, αIL-5) or control Ab (cont IgG1) at day 0 and day 2 post-infection with *N. brasiliensis* (Nb). **(A)** The numbers of worms in the intestine were counted at day 5 after *N. brasiliensis* infection. **(B)** ILC2s, eosinophils, and CD4^+^ cells among the BALF cells were analyzed by flow cytometry (SP6800). Cell populations were defined as follows: ILC2s, FSC^lo^SSC^lo^CD45^+^CD4^−^Lin(CD3, CD8, CD19, NK1.1, IgE, siglec F, Gr-1)^−^Sca-1^+^ST2^+^; Eosinophils, CD45^+^CD11c^lo^CD3^−^B220^−^CCR3^+^; and CD4^+^ cells, FSC^lo^SSC^lo^CD45^+^CD4^+^. **(C)** *Il33, Il13*, and *Epx* mRNA expression levels in the lungs were examined. Data are representative of two independent experiments that had similar results. **(D–F)** *S. venezuelensis*-infected or uninfected (cont) mice (WT, *n* = 7; Δ*dblGATA, n* = 5–6) were infected with *N. brasiliensis*. **(D)** The numbers of worms in the intestine were counted at day 5 after *N. brasiliensis* infection. **(E)** Eosinophils, ILC2s, and CD4^+^ cells among the BALF cells were analyzed as in **(B)**. **(F)** *Il33, Il5*, and *Il13* mRNA expression levels in the lungs were examined. Data are representative of two independent experiments that had similar results.

Since ILC2s are a major source of IL-5 production, we investigated the requirement for IL-5 in the protective phenotype of Sv-exp mice. The blockade of IL-5 completely abolished the protective effect of *S. venezuelensis* pre-infection on *N. brasiliensis* infection (Figure 3A). As expected, the IL-5 depletion strikingly inhibited the accumulation of eosinophils in the BALF from *N. brasiliensis*-infected mice as well as the expression of mRNA for eosinophil peroxidase *(Epx)* in the lungs without affecting the numbers of ILC2s and CD4^+^ T cells or the mRNA expression levels of *Il33* and *Il13* (Figures 3B,C).

To confirm the role of eosinophils, we infected eosinophil-deficient Δ*dblGATA* mice with *S. venezuelensis*, followed later by infection with *N. brasiliensis*. In agreement with the results from the IL-5 neutralization experiment, eosinophil-deficient Sv-exp mice failed to reject *N. brasiliensis* infection, without displaying reductions in the accumulation of ILC2s and CD4^+^ T cells or in the mRNA expression levels of *Il33, Il5*, and *Il13* (Figures 3D–F). These results clearly demonstrate that the enhanced IL-5 production by ILC2s in Sv-exp mice increases the number of eosinophils in the lungs, and these eosinophils contribute to the protection against *N. brasiliensis* infection.

### IL-33 is critical for the resistance of sv-exp mice to infection by *nippostrongylus brasiliensis*

**Figure 4:**
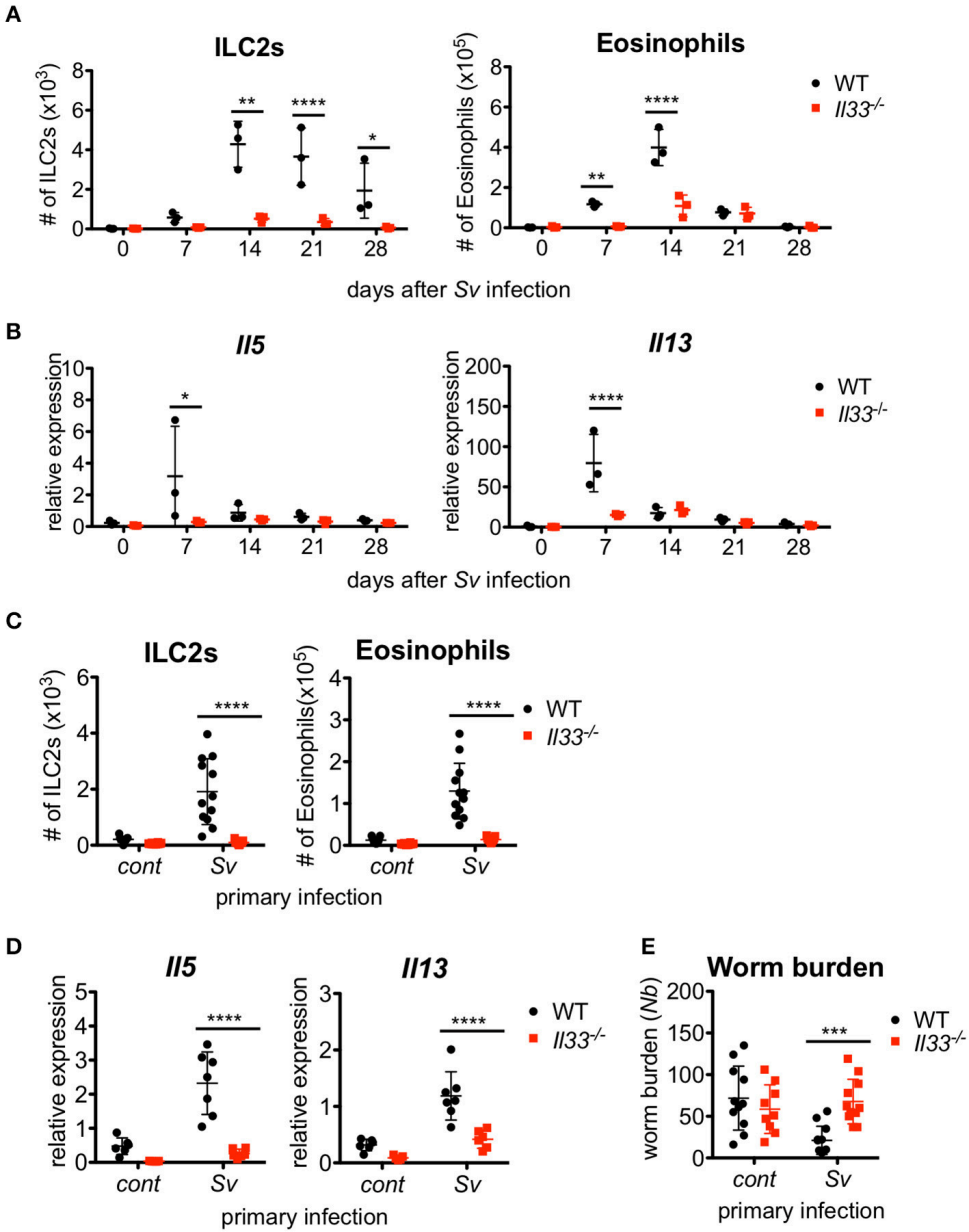
IL-33 is critical for the enhanced inflammation and the protection against *Nippostrongylus brasiliensis* infection. WT and *Il33*^−/−^ mice (*n* = 3) were infected with *S. venezuelensis* (Sv) at day 0. **(A)** BALF cells from uninfected (day 0) or *S. venezuelensis*-infected mice at the indicated days were analyzed as in Figure 1H. Data are representative of two independent experiments that had similar results. **(B)** *Il5* and *Il13* mRNA expression levels in the lungs were examined. **(C–E)** WT and *Il33*^−/−^ mice were infected with *N. brasiliensis* 4 weeks after *S. venezuelensis* (Sv) infection. *S. venezuelensis*-uninfected mice were used as a control (cont). Five days after *N. brasiliensis* infection, the number of ILC2s and eosinophils (*n* = 10–12) **(C)** and the *Il5* and *Il13* mRNA expression levels (*n* = 5–7) in the lungs were examined **(D)**. **(E)** The numbers of worms in the intestine were counted (*n* = 10–12). Pooled data from **(C,E)** or representative of **(D)** two independent experiments are shown (Mean ± SD).

We previously reported that IL-33-deficient mice had an impairment in the early accumulation of eosinophils and ILC2s in response to *S. venezuelensis* infection (31). To determine if the reduction of ILC2s in *Il33*^−/−^ mice was restored by other mechanisms to the level of WT mice after the first 2 weeks post-infection, we infected WT and IL-33-deficient mice with *S. venezuelensis* and analyzed the cell population in the BALF over the following 4 weeks. *Il33*^−/−^ mice were unable to accumulate ILC2s during these 4 weeks (Figure 4A). Although there were significant differences on day(s) 7 and/or 14 in the number of eosinophils and mRNA expression levels of *Il5* and *Il13* between WT and *Il33*^−/−^ mice, by day 21, these differences between WT and *Il33*^−/−^ mice became negligible (Figures 4A,B).

To examine the role of IL-33 in the enhanced lung inflammation induced by subsequent helminth infection, we infected Sv-exp WT and *Il33*^−/−^ mice with *N. brasiliensis*. A lack of IL-33 severely impaired the accumulation of ILC2s and eosinophils along with the induction of cytokine mRNA expression in Sv-exp mice (Figures 4C,D), indicating that IL-33 is required for the accumulation of ILC2s and the severe lung inflammation in sequential nematode infection. Furthermore, *S. venezuelensis* infection-induced resistance to infection by *N. brasiliensis* was abrogated in *Il33*^−/−^ mice (Figure 4E). These data clearly demonstrate the critical role of IL-33 in the enhanced lung inflammation and host defense of Sv-exp mice.

### *Strongyloides venezuelensis* infection induces trained ILC2s

**Figure 5:**
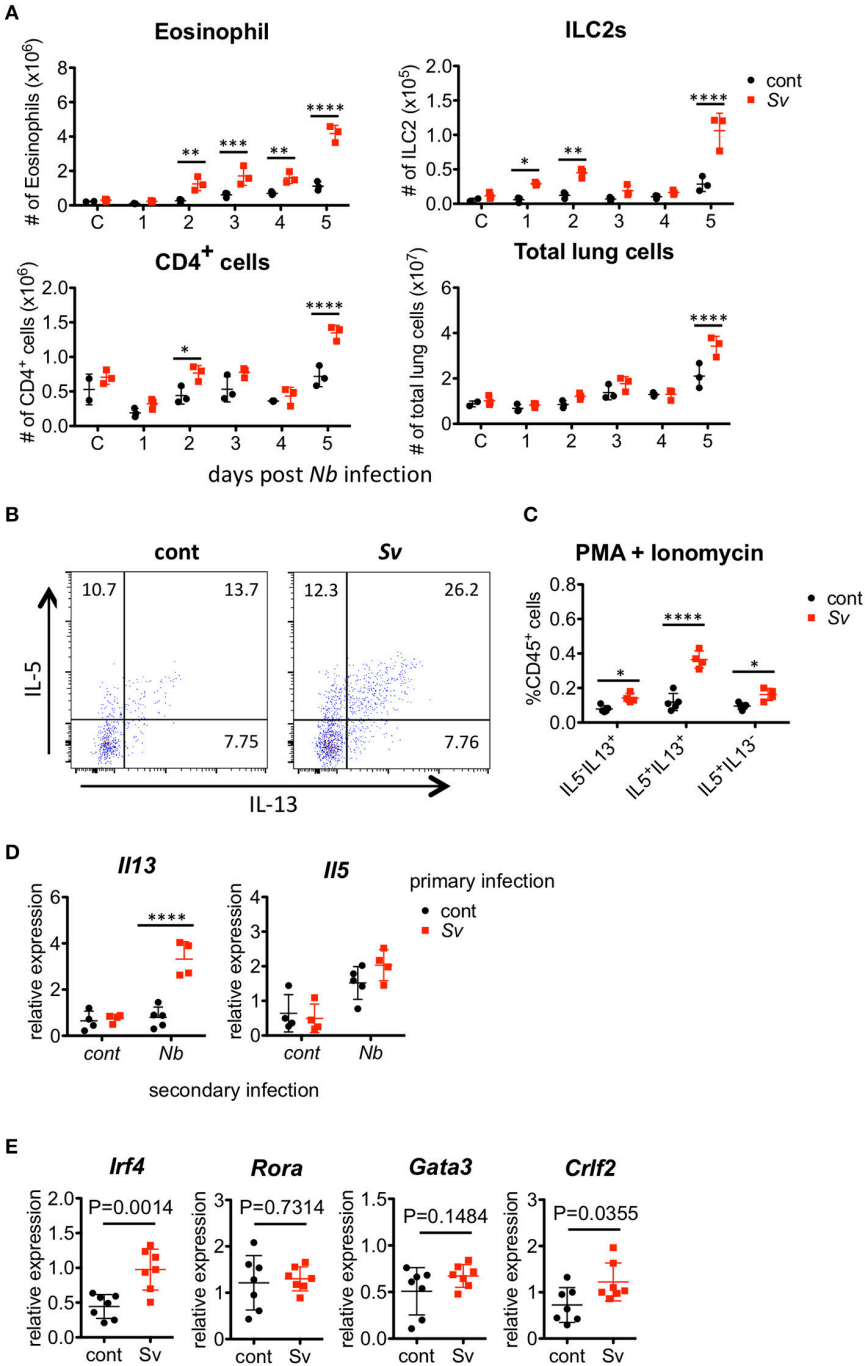
*Strongyloides venezuelensis*-infection induces highly reactive ILC2s in the lungs. **(A)** *S. venezuelensis*-infected (Sv) or uninfected control (cont) mice (*n* = 3) were inoculated with 500 *N. brasiliensis* L3 (Nb). Cell populations of lung cells at the indicated days were analyzed by flow cytometry (SP6800) (Figure S6). **(B)** Eight weeks after *S. venezuelensis* infection, lung cells were stimulated with PMA and ionomycin for 3 h in the presence of brefeldin A. Intracellular IL-5 and IL-13 in ILC2s were analyzed by flow cytometry (SP6800). ILC2s were gated on Fixable Viability Dye^−^CD45^+^Lin(CD3, CD4, CD8, CD11b, B220, NK1.1, IgE, Gr-1)^−^ST2^+^Sca-1^+^Thy1.2^+^ cells. **(C)** The proportions of IL-5^+^IL-13^−^, IL-5^−^IL-13^+^, and IL-5^+^IL-13^+^ ILC2s in the population of CD45^+^ cells (*n* = 4–5). **(D)** Lung ILC2s were sorted from *S. venezuelensis*-infected or uninfected mice (*n* = 4–5) before or 2 days after infection with *N. brasiliensis* L3. The expression levels of *Il5* and *Il13* mRNA were analyzed by Q-PCR. Data are representative of two independent experiments that had similar results (Mean ± SD). **(E)** The expression levels of transcription factors and TSLPR *(Crlf2)* in lung ILC2s were analyzed by Q-PCR. Statistical analyses were performed using Student's *t*-tests. Data are representative of two independent experiments (Mean ± SD).

*N. brasiliensis* larvae enter the lung around day 2 after they are subcutaneously injected into a host, and they migrate to the intestine around day 5 (38); therefore, early accumulation and activation of ILC2s and eosinophils in the lungs may be important for the nematode infection resistance of Sv-exp mice. To examine the timing of the inflammatory cell increase in the lungs after *N. brasiliensis* infection, we analyzed the lung cells of Sv-exp mice and control mice daily. We found that the numbers of pulmonary eosinophils, ILC2s, and CD4^+^ T cells increased in Sv-exp mice on day 2 post-infection, whereas these cell populations did not increase significantly in control mice (Figure 5A and (Figure S6). Because the early eosinophil accumulation could potentially be due to an increased responsiveness of ILC2s rather than only an increase in the number of these cells, next we compared the reactivity of ILC2s from the lungs of Sv-exp and control mice. The experience of *S. venezuelensis* infection significantly increased the proportion of IL-5/IL-13 single- and double-positive lung ILC2s in response to PMA and ionomycin stimulation (Figures 5B,C), indicating that ILC2s from Sv-exp mice have more cytokine-producing capacity compared with those from control mice.

We also analyzed the cytokine expressions in sorted ILC2s from the lungs of control mice or Sv-exp mice 2 days after *N. brasiliensis* infection. *N. brasiliensis* infection clearly increased the Il13 mRNA expression in ILC2s from Sv-exp mice compared with that in ILC2s from control mice (Figure 5D), indicating that the ILC2s in Sv-exp mouse lungs responded promptly and strongly to *N. brasiliensis* infection *in vivo*. To investigate the differences between ILC2s from Sv-exp and control mice, we analyzed the expression of various transcription factors and surface markers (Figure 5E) and (Figure S7). We found that the expression level of *Irf4* mRNA was significantly higher in ILC2s from Sv-exp mice compared with control mice, whereas the mRNA levels of *Rora* and *Gata3*, which are critical transcription factors for ILC2 development and function (23–25), were not different. The expression level of mRNA for TSLPR *(Crlf2)* was also enhanced in Sv-exp ILC2s compared with control ILC2s. We did not detect any enhanced surface expression of IL-25R or ST2, suggesting that the enhanced ILC2 response was not caused by a change in the expression of receptors for IL-25 and IL-33. Together, these results demonstrate that the ILC2s in Sv-exp mice not only increased their number in the lungs but also changed their phenotype to that of highly responsive ILC2s. Since these ILC2s have been trained by *S. venezuelensis* infection to acquire a highly responsive phenotype, we call these cells “trained ILC2s.”

### IL-33 is not sufficient to induce trained ILC2s or protective immunity against *nippostrongylus brasiliensis* infection

**Figure 6:**
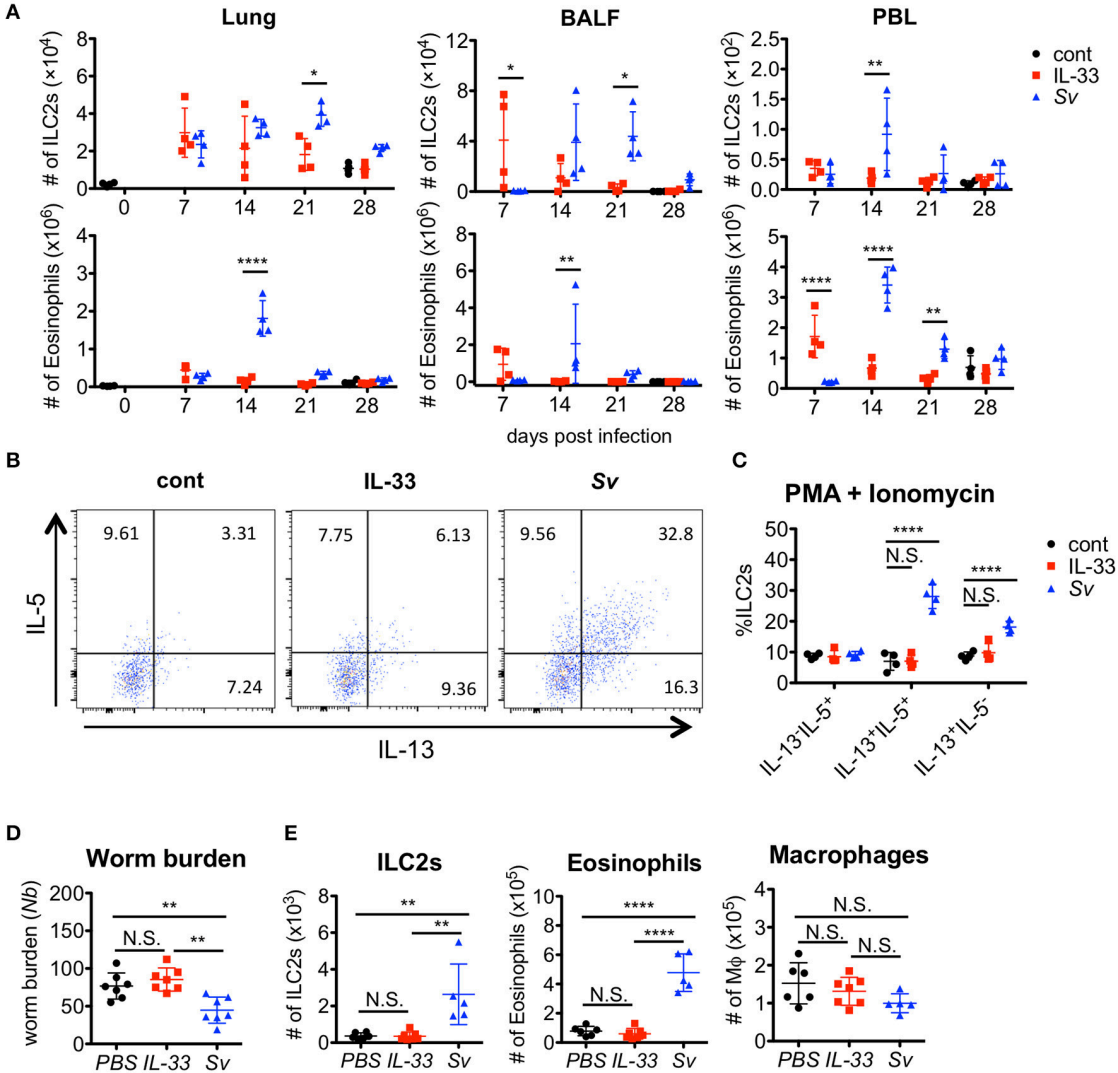
IL-33 is not sufficient for the induction of trained ILC2s. WT C57BL/6 mice were subcutaneously inoculated with 5000 *S. venezuelensis* L3 at day 0 *(Sv)* or were intranasally administrated 100 ng of IL-33 at days 0, 1, 2, and 3 (IL-33). cont, untreated control. **(A)** Cell populations in the lung cells, BALF cells, and peripheral blood leukocytes (PBLs) at indicated days were analyzed by flow cytometry (SP6800) (*n* = 4). Cell populations were defined as follows: Eosinophils, PI^−^CD45^+^CD11c^int^CD3^−^B220^−^CCR3^+^ and ILC2s, PI^−^FSC^lo^SSC^lo^CD45^+^Thy1.2^+^Lin^−^Sca-1^+^ST2^+^. **(B)** Four weeks after treatment, lung cells were stimulated with PMA and ionomycin for 3 h in the presence of brefeldin A. Intracellular levels of IL-5 and IL-13 in ILC2s were analyzed by flow cytometry (SP6800) as in Figure S8. **(C)** The proportions of IL-5^+^IL-13^−^, IL-5^+^IL-13^+^, and IL-5^−^IL-13^+^ cells within the population of ILC2s (*n* = 4). **(D,E)** Mice were infected with *N. brasiliensis* 4 weeks after *S. venezuelensis* infection or IL-33 treatment. Control mice were treated with PBS (PBS) instead of IL-33. Five days after *N. brasiliensis* infection, **(D)** the numbers of worms in the intestine were counted (*n* = 7). **(E)** The numbers of ILC2s, eosinophils, and macrophages (*n* = 5–7) among the BALF cells were examined. Statistical analyses were performed using a two-way ANOVA **(A,C)** or one-way ANOVA **(D)** with Bonferroni *post-hoc* tests. Data are representative of three independent experiments (Mean ± SD).

We and other groups previously reported that IL-33 has a strong capacity to induce the proliferation and activation of ILC2s (31, 40, 41, 44). To determine if IL-33 is sufficient for the induction and retention of trained ILC2s in the lung and for protection against *N. brasiliensis* infection, we administered IL-33 intranasally into WT C57BL/6 mice and analyzed various populations in the BALF cells weekly. An accumulation of ILC2s was induced promptly and intensively in IL-33-treated mice at day 7. At day 21, when the number of nematode infection-induced ILC2s reached their population peak, the number of IL-33-induced ILC2s decreased. Finally, the number of ILC2s in IL-33-treated mice returned to the level of phosphate-buffered saline (PBS)-treated control mice at day 28 (Figure 6A).

To examine the capacity of ILC2s to produce cytokines, lung cells from PBS-treated, IL-33-treated, or Sv-exp mice were stimulated with PMA + ionomycin. *S. venezuelensis* infection induced hyperreactive ILC2s, whereas IL-33 treatment failed to enhance the responsiveness of ILC2s (Figures 5B,C, 6B,C). Similar results were also obtained following cytokine (IL-7 + IL-25 + IL-33) stimulation (Figure S8). Furthermore, IL-33-treated mice failed to be protected against *N. brasiliensis* infection (Figure 6D) and failed to show accumulations of ILC2s and eosinophils in the lungs (Figure 6E). These differences following *S. venezuelensis* infection and IL-33 treatment suggest that the training of ILC2s requires some other stimuli in addition to IL-33.

## Discussion

Here, we clarified the immune modification effect of *S. venezuelensis* infection on a subsequent *N. brasiliensis* infection and determined its mechanism. Our data demonstrate that a host suffering from *S. venezuelensis* exposure is resistant to a subsequent *N. brasiliensis* infection. Our results showed that IL-33 as well as lung ILC2s and eosinophils are necessary for this resistance. Furthermore, we found that the lung ILC2s of Sv-exp mice gained a “trained” phenotype and had a rapid and high reactivity against *N. brasiliensis* infection. Together, our findings suggest that a host may be resistant to subsequent helminth infection within the weeks following a primary infection with other pulmonary migratory nematode due to the production of high cytokine levels by trained ILC2s waiting in the lung, which induce a rapid accumulation of eosinophils in response to IL-33.

Since mice that have been infected with *S. venezuelensis* develop immune memory, most *S. venezuelensis* larvae cannot migrate to the lungs during reinfection attempts (39). In agreement with previous findings, reinfection of mice by *S. venezuelensis* induced only weak *Il33* mRNA expression compared with primary infection and was unable to induce Th2 cytokine expression despite the relatively high numbers of ILC2s remaining in the lungs of Sv-exp mice.

It is well-known that the main components of immune memory are antigen-specific T cells and B cells, but, in some cases, natural killer (NK) cells also can be antigen-specific memory cells (45). For example, antigen-specific memory NK cells have the capacity to protect DAP12-deficient neonatal mice from mouse cytomegalovirus infection (46). Furthermore, stimulation with the cytokines IL-12, IL-15, and IL-18 can induce long-lasting non-antigen-specific NK cells (47). In addition to NK cells, it has recently been clarified that myeloid cells, such as macrophages, have a mechanism to remediate pathogen exposure (48, 49). These phenomena are described as “trained immunity” or “innate immune memory.”

We found that Sv-exp mice acquired specific-antigen-independent immunity; specifically, mice that experienced a *S. venezuelensis* infection gained significantly higher resistance to infection by *N. brasiliensis*. Similarly, mice that experienced a *N. brasiliensis* infection become resistant to infection by *S. venezuelensis*; our findings are consistent with a previous report by Baek et al. which suggested the contribution of eosinophils to this process (50). In the present study, we demonstrated the critical role of eosinophils in the resistance to subsequent infection by a different species of parasitic worm, and we further showed that this protection depends on IL-33 and ILC2s, but not on CD4^+^ cells, suggesting that mice acquired CD4-independent “innate immune memory” or “trained immunity” following infection with *S. venezuelensis*. As the cytotoxic activity of eosinophils against *N. brasiliensis* larvae has already been demonstrated (51–53), it seems likely that the reduction in the number of worms in the small intestine is due to larval injury by eosinophils. However, we cannot completely exclude the contribution of acquired immunity to this protection because antibodies produced in response to the first infection may possibly bind to the larvae of *N. brasiliensis* via antigen cross-reaction.

Our data suggest that the lung is an important site for the resistance to subsequent nematodes that use this organ in their life cycle. Previous work showed that macrophages activated by CD4^+^ T cells and ILC2s injure larvae in the lungs upon reinfection by *N. brasiliensis* (54). Additionally, neutrophils produce IL-13 and induce macrophages to develop into M2 macrophages, which are long-lived macrophages with parasite killing activity (55). Long-lived macrophages may remain in the lungs of *S. venezuelensis*-infected mice. Since eosinophils also have the ability to produce IL-4 and IL-13 (56, 57), it is possible that the final effector cells are macrophages. However, at least in our experimental system, ILC2s and eosinophils were found to be essential for host defense against a second infection.

Guo et al. reported that Ascaris suum-infected mice were resistant to *N. brasiliensis* infection due to the activation of antigen-independent CD4^+^ Th2 cells by IL-33 stimulation (58). In their experiments, the mice were re-infected early after the first infection (day 11), and accumulated Th2 cells from the initial infection were likely to respond to the next infection. In contrast, our model examined infection after a longer period post-initial infection (day 28), and it seems that the contribution of ILC2s becomes higher than that of CD4^+^ T cells when the second infection occurs later.

During the initial infection with *S. venezuelensis*, the numbers of ILC2s and eosinophils in the lung increase after day 7 of infection. However, larvae pass through the lungs from day 1 to day 4 of infection (36), so eosinophils would need to accumulate in the lungs within 4 days for these cells to attack lung larvae. Here, we demonstrated that the number of eosinophils increased in Sv-exp mice beginning from day 2 of *N. brasiliensis* infection. This rapid increase in eosinophils is probably due to the increased number of ILC2s resulting from prior *S. venezuelensis* infection along with the ability of ILC2s to rapidly and strongly respond to *N. brasiliensis* infection.

IL-33, a critical cytokine for activation of ILC2s, is expressed in many types of cells including epithelial and endothelial cells and released in response to necrotic damages (59). In the lung, type II alveolar epithelial cells possess IL-33 even in the normal state and increase its expression by nematodes infection (31). Here we demonstrated IL-33 is actually essential for the activation of ILC2s to accumulate eosinophils. However, as the IL-33 expression before and after *N. brasiliensis* infection is comparable between control and Sv-exp mice, the increase of IL-33 expression is transient and the previous responses do not appear to be memorized.

Martinez-Gonzalez and colleagues reported that IL-33 induces memory ILC2s (60). They showed that intranasal treatment with IL-33 or papain induces memory ILC2s in mediastinal lymph nodes in mice, and these cells respond strongly to subsequent challenge with papain or IL-33, respectively. In our study, IL-33 was essential for the induction of highly reactive ILC2s; however, following IL-33 administration, the ILC2s in the lungs disappeared promptly after a transient proliferation. This result is consistent with their report that memory ILC2s are not in the lungs. However, highly reactive ILC2s are present in the lungs of *S. venezuelensis*-infected mice, and they respond strongly to *N. brasiliensis* infection. Nevertheless, when we infected IL-33-treated mice with *N. brasiliensis*, we did not observe the resistance to subsequent nematode infection and increases in pulmonary ILC2s and eosinophils that occurred in Sv-exp mice. There are two differences in the experimental design between these two studies. First, we used a dose of IL-33 capable of inducing the same ILC2 level as that induced by nematode infection, which is lower than the dose used in the prior work. Second, different materials were used as the challenge. We employed a nematode as the challenge instead of papain. Our results suggest that the activation of ILC2s requires IL-33 but the induction of trained ILC2s likely requires some factors other than IL-33.

We found that ILC2s in Sv-exp mice showed enhanced expression of mRNA for TSLPR and IRF4. Mohapatra et al. reported that *N. brasiliensis* infection induced TSLP release in the BALF at 25 h and that TSLP/IL-33 stimulation of ILC2s induced the production of Th2 cytokines in an IRF4-dependent manner (61), suggesting the enhanced function of ILC2s in Sv-exp mice is due to their increased expression of these molecules. They also demonstrated that stimulation of TSLP with IL-33 induced IL-9 production by ILC2s (61). IL-9 has been shown to act autocrinally to maintain lung ILC2s and to play a role in tissue repair after infection with *N. brasiliensis* (29). Additional work is needed to determine why ILC2s remain for a long time after *S. venezuelensis* infection and why they show enhanced reactivity.

In conclusion, we have shown that *S. venezuelensis*-infected hosts acquire specific-antigen-independent “trained immunity” that contributes to a substantial resistance to pulmonary migratory nematodes. This is beneficial for host animals that live in areas where there is more than one parasitic infestation.

## Author contributions

KY, AK, and TA carried out the experiments. KY and KN planned the experiments and supervised the study. KY wrote the manuscript. KY, TA, and KN analyzed the data.

### Conflict of interest statement

The authors declare that the research was conducted in the absence of any commercial or financial relationships that could be construed as a potential conflict of interest.

## Abbreviations

Ab: Antibody
BALF: Bronchoalveolar lavage fluid
GATA3: GATA-binding protein 3
ILC2: Group 2 innate lymphoid cell
L3: Third stage larva
NK: Natural killer
PBL: Peripheral blood leukocyte
Q-PCR: Quantitative PCR
RORα: Retinoic acid receptor-related orphan receptor alpha
Sv-exp: *Strongyloides venezuelensis* experienced
TSLP: Thymic stromal lymphopoietin.
