# Supplementary material for: Nematode-Infected Mice Acquire Resistance to Subsequent Infection With Unrelated Nematode by Inducing Highly Responsive Group 2 Innate Lymphoid Cells in the Lung
Source: Front Immunol. 2018 Sep 19;9:2132. doi: 10.3389/fimmu.2018.02132 (PMC6157322; doi:10.3389/fimmu.2018.02132)
Supplement: Supplementary file 1 [file Data_Sheet_1.PDF]

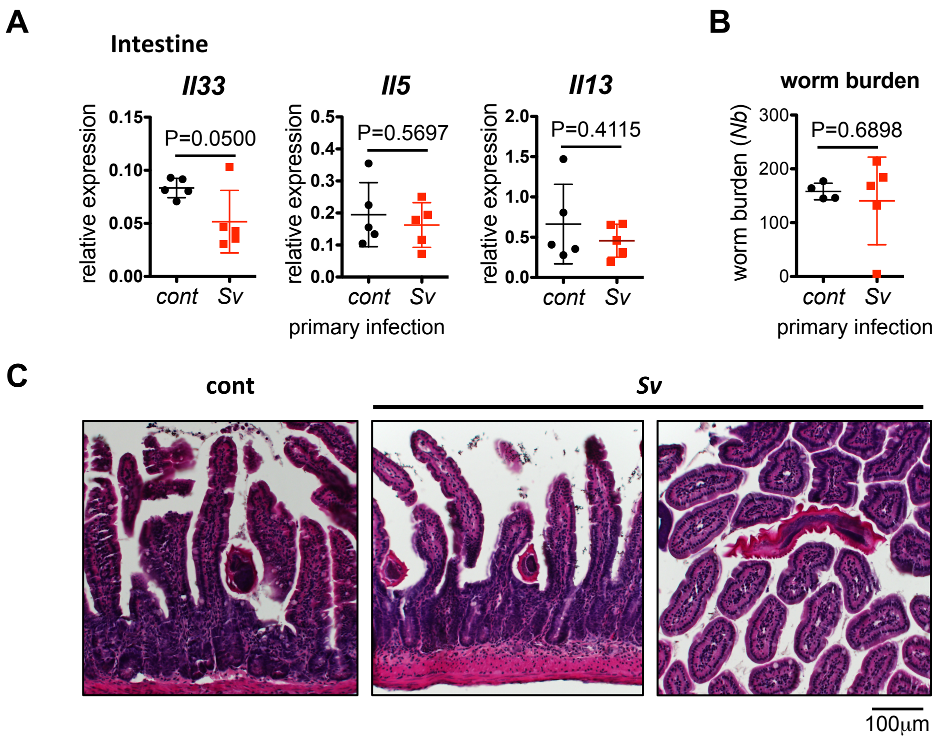

Figure S1. The immunological status of the intestine was not affected by *Strongyloides venezuelensis* infection.

(A) *S. venezuelensis*-infected (*Sv*) or uninfected control (cont) mice were inoculated with *N. brasiliensis* (*Nb*) 4 weeks later. Five days after *N. brasiliensis* infection, the levels of *Il33, Il5* and *Il13* mRNA expression in the intestines were examined. **(B)** The number of *N. brasiliensis* adult worms from *S. venezuelensis*-experienced or control mice (n = 5). *N. brasiliensis* adult worms were transferred into the intestines of mice 4 weeks after primary *S. venezuelensis* infection. Sixteen hours after *N. brasiliensis* transfer, attached adult worms were isolated from the intestines and counted. **(C)** Histological analysis. *N. brasiliensis* adult worms were transferred into the intestines of mice 10 weeks after primary *S. venezuelensis* infection. Sixteen hours after transfer, jejunum specimens were stained with hematoxylin and eosin. Scale bar: 100 μm. Statistical analyses were performed using Student's *t*-tests. Data are representative of two independent experiments (Mean ± SD).

Figure S1
