# Supplementary material for: Nematode-Infected Mice Acquire Resistance to Subsequent Infection With Unrelated Nematode by Inducing Highly Responsive Group 2 Innate Lymphoid Cells in the Lung
Source: Front Immunol. 2018 Sep 19;9:2132. doi: 10.3389/fimmu.2018.02132 (PMC6157322; doi:10.3389/fimmu.2018.02132)
Supplement: Supplementary file 2 [file Data_Sheet_2.PDF]

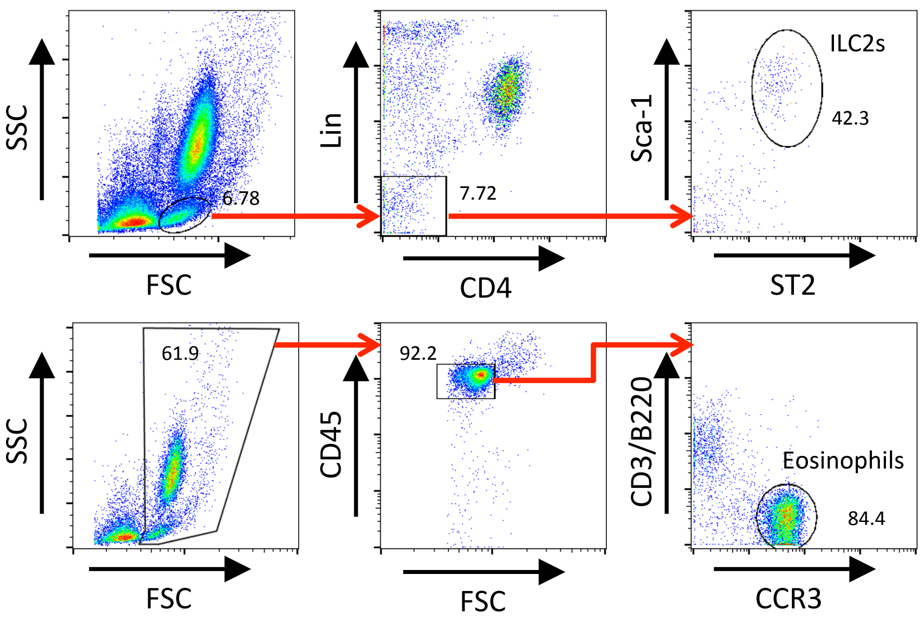

Figure S2. The gating strategy used to quantify BALF cells in Fig. 1.
Cell populations among BALF cells were analyzed by flow cytometry (FACScalibur) and defined as follows: ILC2s, $FSC^{lo}SSC^{lo}Lin^{-}Sca\text{-}1^{+}ST2^{+}$; Eosinophils, $CD45^{+}CD3^{-}B220^{-}CCR3^{+}$; CD3/B220, $CD45^{+}CCR3^{-}CD3^{+}B220^{+}$; and Monocytes, $CD45^{+}Autofluorescence^{high}$.

Figure S2
