# Supplementary material for: Nematode-Infected Mice Acquire Resistance to Subsequent Infection With Unrelated Nematode by Inducing Highly Responsive Group 2 Innate Lymphoid Cells in the Lung
Source: Front Immunol. 2018 Sep 19;9:2132. doi: 10.3389/fimmu.2018.02132 (PMC6157322; doi:10.3389/fimmu.2018.02132)
Supplement: Supplementary file 3 [file Data_Sheet_3.PDF]

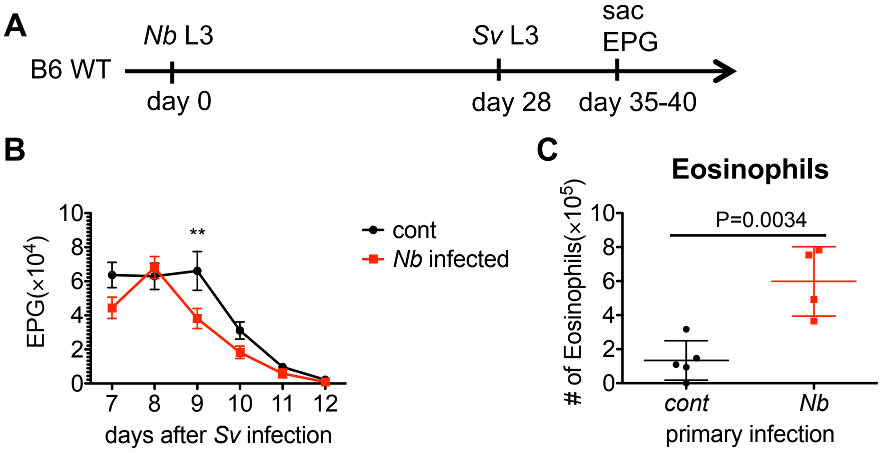

Figure S3. *Nippostrongylus brasiliensis*-experienced mice develop resistance against *Strongyloides venezuelensis* infection.

(A) Experimental workflow for sequential nematode infection. Seven-week-old C57BL/6 (B6) mice were infected with *N. brasiliensis* (*Nb*) or uninfected at day 0. twenty-eight days later, mice were inoculated with 500 *S. venezuelensis* (*Sv*) L3. sac; sacrificed. **(B)** Kinetics of the number of eggs per gram feces (EPG) at the indicated days. Results are geometric means +/− SEM (n = 6–7). **, $p < 0.01$ versus the corresponding value for control mice (Two-way ANOVA with Bonferroni post-hoc tests). **(C)** The number of eosinophils in the BALF at 7 days after *S. venezuelensis* infection was analyzed by flow cytometry as in **Fig. S2**. cont; control. Statistical analyses were performed using Student's *t*-tests. Data are representative of two independent experiments (Mean ± SD).

Figure S3
