# Supplementary material for: Nematode-Infected Mice Acquire Resistance to Subsequent Infection With Unrelated Nematode by Inducing Highly Responsive Group 2 Innate Lymphoid Cells in the Lung
Source: Front Immunol. 2018 Sep 19;9:2132. doi: 10.3389/fimmu.2018.02132 (PMC6157322; doi:10.3389/fimmu.2018.02132)
Supplement: Supplementary file 4 [file Data_Sheet_4.PDF]

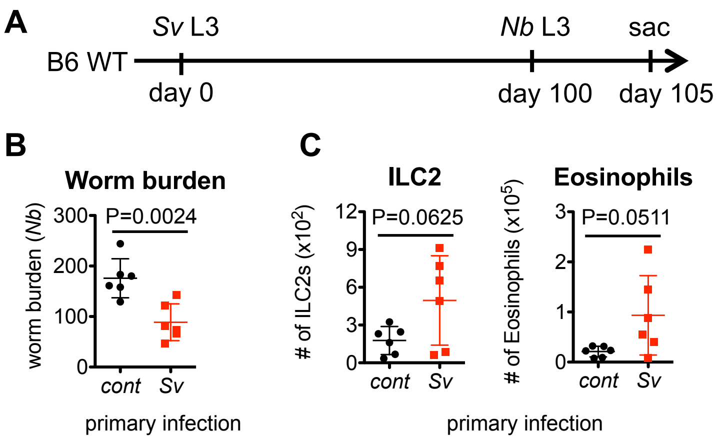

Figure S4. The effect of experiencing a *Strongyloides venezuelensis* infection lasts at least 3 months.

(A) Experimental workflow for sequential nematode infection. Mice (n = 6) were inoculated with 500 *N. brasiliensis* (*Nb*) L3 at 100 days after *S. venezuelensis* (*Sv*) infection. sac; sacrificed. Five days after *N. brasiliensis* infection, (**B**) the numbers of worms in the intestine were counted. cont; control. (**C**) The numbers of ILC2s and eosinophils among the BALF cells were analyzed by flow cytometry (SP6800). Statistical analyses were performed using Student's *t*-tests. Data are representative of two independent experiments (Mean ± SD).

Figure S4
