# Supplementary material for: Nematode-Infected Mice Acquire Resistance to Subsequent Infection With Unrelated Nematode by Inducing Highly Responsive Group 2 Innate Lymphoid Cells in the Lung
Source: Front Immunol. 2018 Sep 19;9:2132. doi: 10.3389/fimmu.2018.02132 (PMC6157322; doi:10.3389/fimmu.2018.02132)
Supplement: Supplementary file 5 [file Data_Sheet_5.PDF]

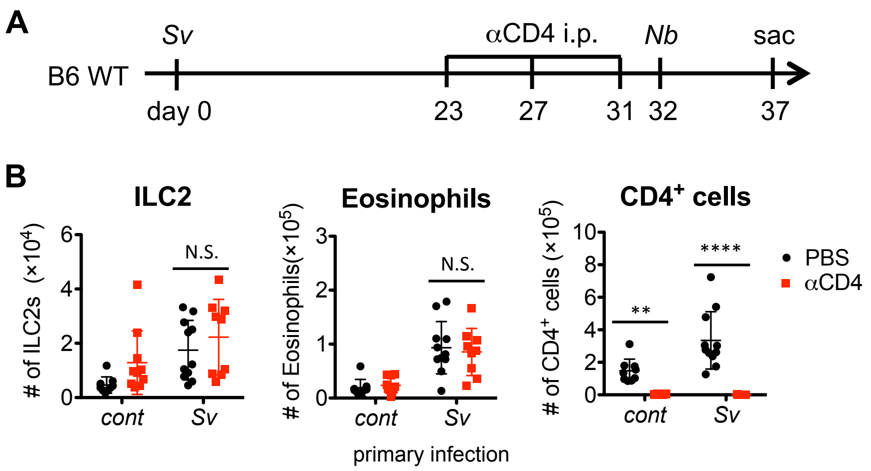

Figure S5. $CD4^+$ cells are dispensable for the enhanced inflammation following *Strongyloides venezuelensis* infection.

(**A**) Experimental workflow for sequential nematode infection. Mice were inoculated with 5000 *S. venezuelensis* (*Sv*) L3 at day 0, followed by *N. brasiliensis* (*Nb*) infection 4 weeks later. Mice were treated with anti-CD4 Ab αCD4, 500 μg) or PBS at 1, 5, and 9 days before *N. brasiliensis* infection. sac; sacrificed. (**B**) ILC2s and eosinophils among the BALF cells were analyzed by flow cytometry (LSRFortessa) (n = 10–11), and the cell populations were defined as follows: ILC2s, $FSC^{lo}SSC^{lo}CD45^+CD4^-Lin^-$ $Sca\text{-}1^+ST2^+$; Eosinophils, $CD45^+CD11c^{lo}CD3^-B220^-CCR3^+$; and CD4, $FSC^{lo}SSC^{lo}CD45^+Lin^+CD4^+$. cont; control. Pooled data from two independent experiments are shown (mean ± SD).

Figure S5
