# Supplementary material for: Nematode-Infected Mice Acquire Resistance to Subsequent Infection With Unrelated Nematode by Inducing Highly Responsive Group 2 Innate Lymphoid Cells in the Lung
Source: Front Immunol. 2018 Sep 19;9:2132. doi: 10.3389/fimmu.2018.02132 (PMC6157322; doi:10.3389/fimmu.2018.02132)
Supplement: Supplementary file 6 [file Data_Sheet_6.PDF]

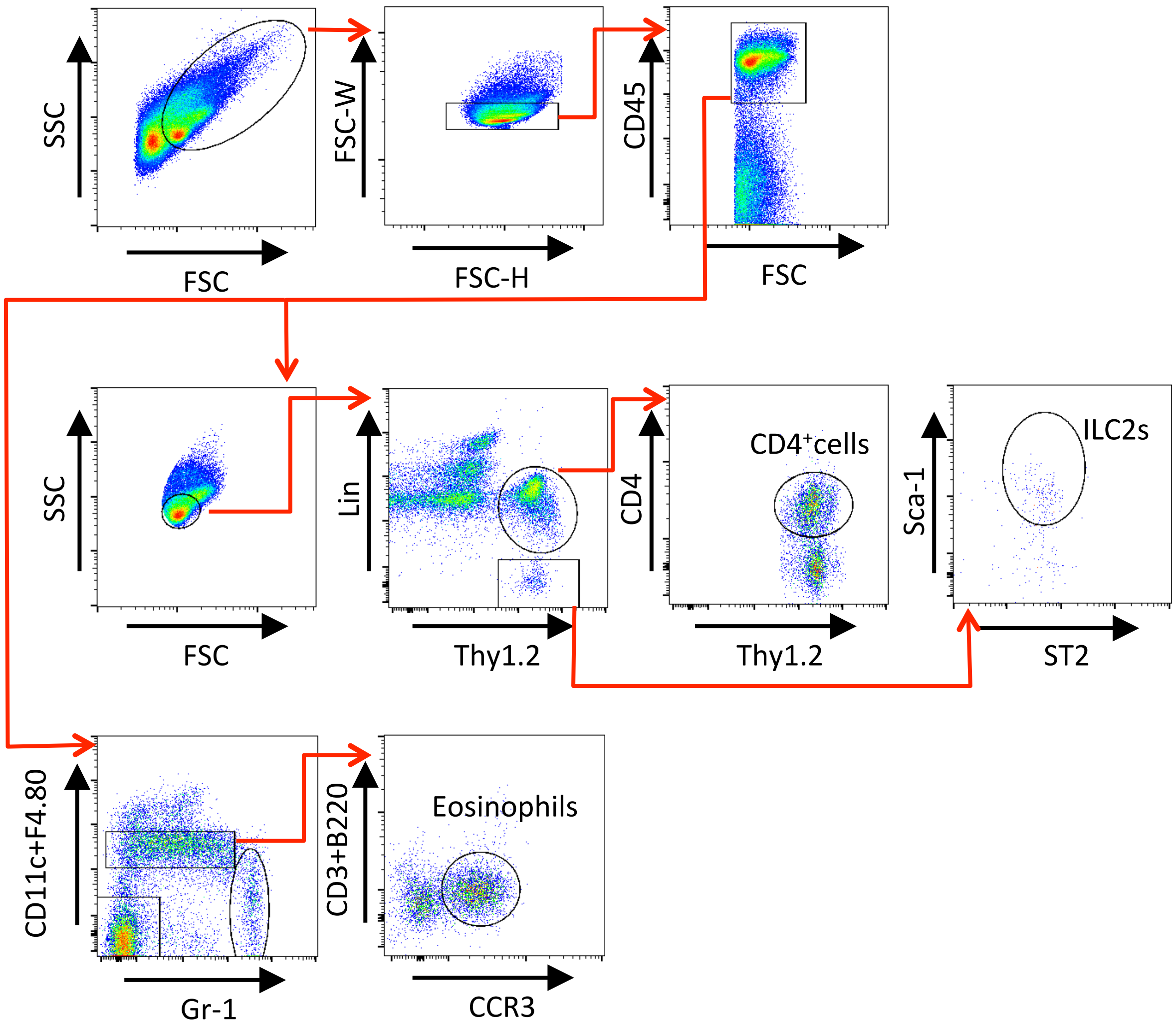

Figure S6. Gating strategy to quantify lung inflammatory cells in Figure 5. Cell populations of lung cells were analyzed by flow cytometry (SP6800) and defined as follows: Eosinophils, $CD45^{+}CD11c^{int}CD3^{-}B220^{-}CCR3^{+}$; ILC2s, $FSC^{lo}SSC^{lo}CD45^{+}Thy1.2^{+}Lin^{-}Sca\text{-}1^{+}ST2^{+}$; and $CD4^{+}$ cells, $FSC^{lo}SSC^{lo}CD45^{+}Lin^{+}Thy1.2^{+}CD4^{+}$.

Figure S6
