# Supplementary material for: Nematode-Infected Mice Acquire Resistance to Subsequent Infection With Unrelated Nematode by Inducing Highly Responsive Group 2 Innate Lymphoid Cells in the Lung
Source: Front Immunol. 2018 Sep 19;9:2132. doi: 10.3389/fimmu.2018.02132 (PMC6157322; doi:10.3389/fimmu.2018.02132)
Supplement: Supplementary file 7 [file Data_Sheet_7.PDF]

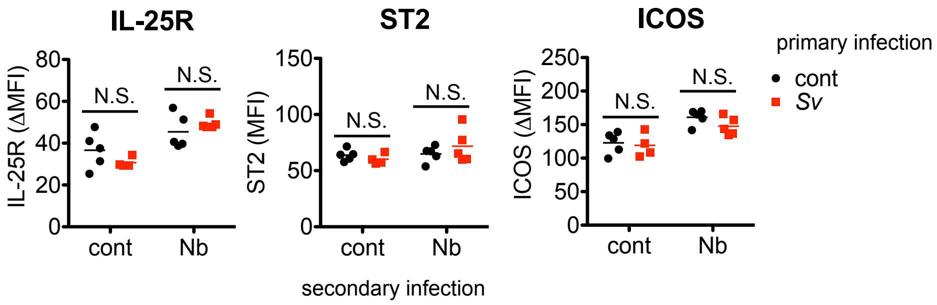

Figure S7. Flow cytometric analysis of surface molecules on ILC2s.
The expression levels of IL-25R, ICOS, and ST2 on lung ILC2s were analyzed by flow cytometry (SP6800). Live $CD45^{+}Lin^{-}Thy1^{+}Sca\text{-}1^{+}ST2^{+}$ cells were gated as ILC2s. cont; control, *Sv*; *S. venezuelensis*. *Nb*; *N. brasiliensis*. Statistical analyses were performed using two-way ANOVAs with Bonferroni post-hoc tests.

Figure S7
