# Supplementary material for: Nematode-Infected Mice Acquire Resistance to Subsequent Infection With Unrelated Nematode by Inducing Highly Responsive Group 2 Innate Lymphoid Cells in the Lung
Source: Front Immunol. 2018 Sep 19;9:2132. doi: 10.3389/fimmu.2018.02132 (PMC6157322; doi:10.3389/fimmu.2018.02132)
Supplement: Supplementary file 8 [file Data_Sheet_8.PDF]

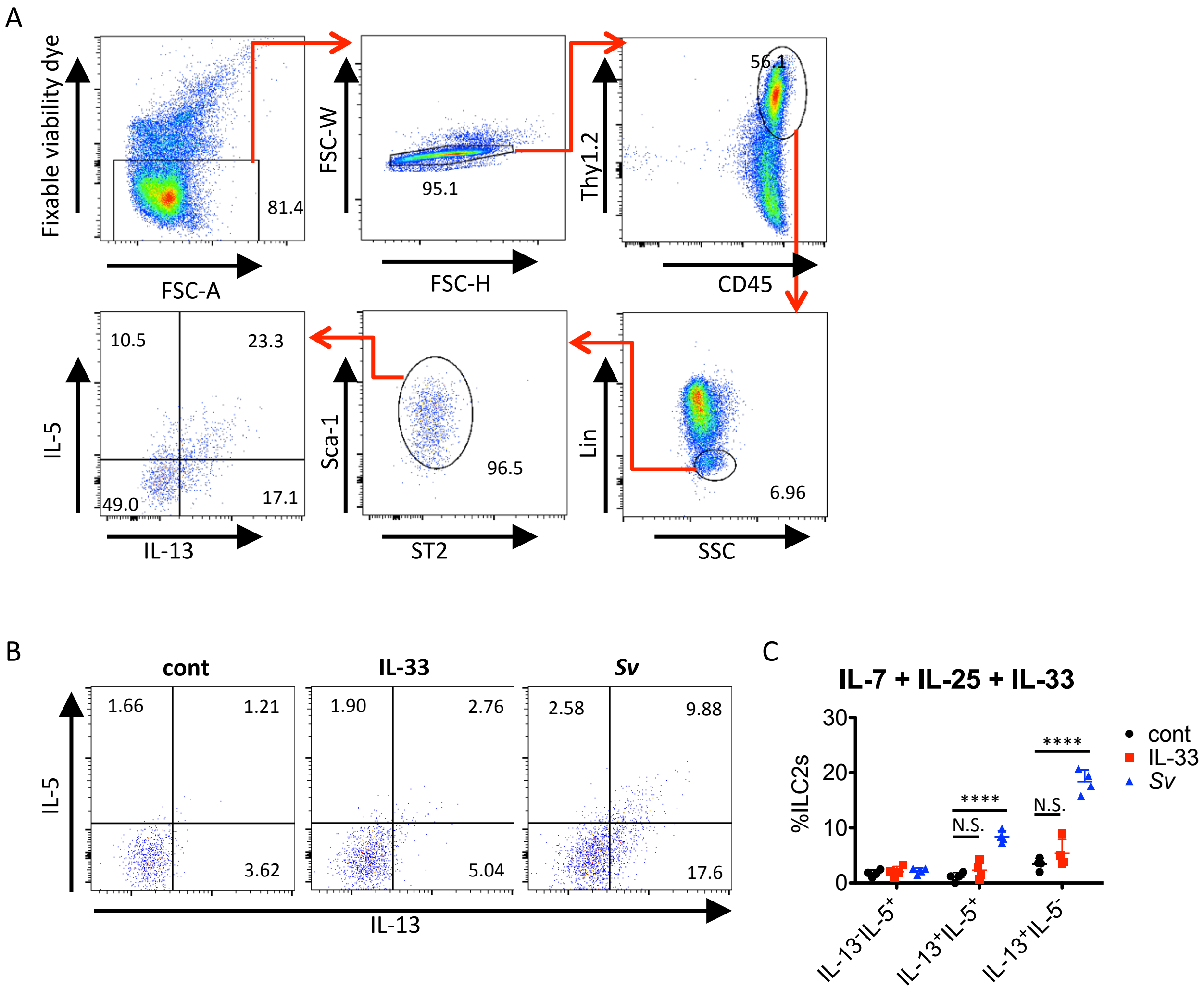

Figure S8. IL-33 is not sufficient for the induction of trained ILC2s in the lung.
(A) The gating strategy for intracellular cytokine staining in **Fig. 6.** Lung leukocytes were stimulated with PMA and ionomycin in the presence of brefeldin A for 4 h or cytokines for 24 h (brefeldin A was added for only the last 6 h). Cells were stained with fixable viability dye eFluor 506 followed by surface markers, after which they were fixed, permeabilized, and stained for intracellular cytokines. Cell populations of lung cells were analyzed by flow cytometry (SP6800), and ILC2s were defined as Viability dye^{-}CD45^{+}Thy1.2^{+}Lin^{-}Sca-1^{+} ST2^{+} cells. (**B**) Four weeks after treatment as in Fig. 6, lung cells were stimulated with IL-7, IL-25, and IL-33 for 24 h (brefeldin A was added for the last 6 h). Levels of intracellular IL-5 and IL-13 in ILC2s were analyzed by flow cytometry (SP6800). cont; control, *Sv*; *S.venezuelensis*. (**C**) The proportions of IL-5^{+}IL-13^{-}, IL-5^{+}IL-13^{+}, and IL-5^{-}IL-13^{+} cells in the population of ILC2s (n = 4). Statistical analyses were performed using two-way ANOVAs with Bonferroni post-hoc tests. Data are representative of two independent experiments (Mean ± SD).

Figure S8
